# Topoisomerase IV is required for partitioning of circular chromosomes but not linear chromosomes in *Streptomyces*

**DOI:** 10.1093/nar/gkt757

**Published:** 2013-08-31

**Authors:** Tzu-Wen Huang, Chin-Chen Hsu, Han-Yu Yang, Carton W. Chen

**Affiliations:** Department of Life Sciences and Institute of Genome Sciences, National Yang-Ming University, Shih-Pai 112, Taiwan

## Abstract

Filamentous bacteria of the genus *Streptomyces* possess linear chromosomes and linear plasmids. Theoretically, linear replicons may not need a decatenase for post-replicational separation of daughter molecules. Yet, *Streptomyces* contain *parC* and *parE* that encode the subunits for the decatenase topoisomerase IV. The linear replicons of *Streptomyces* adopt a circular configuration *in vivo* through telomere–telomere interaction, which would require decatenation, if the circular configuration persists through replication. We investigated whether topoisomerase IV is required for separation of the linear replicons in *Streptomyces*. Deletion of *parE* from the *Streptomyces coelicolor* chromosome was achieved, when *parE* was provided on a plasmid. Subsequently, the plasmid was eliminated at high temperature, and Δ*parE* mutants were obtained. These results indicated that topoisomerase IV was not essential for *Streptomyces*. Presumably, the telomere–telomere association may be resolved during or after replication to separate the daughter chromosomes. Nevertheless, the mutants exhibited retarded growth, defective sporulation and temperature sensitivity. In the mutants, circular plasmids could not replicate, and spontaneous circularization of the chromosome was not observed, indicating that topoisomerase IV was required for decatenation of circular replicons. Moreover, site-specific integration of a plasmid is impaired in the mutants, suggesting the formation of DNA knots during integration, which must be resolved by topoisomerase IV.

## INTRODUCTION

Most bacterial chromosomes consist of covalently closed circular DNA with negative superhelicity. Two counteracting topoisomerases, gyrase and topoisomerase I (Topo I), are responsible for the maintenance of balanced negative superhelicity of these circular DNA molecules. Gyrase, a GyrA_2_GyrB_2_ heterotetramer, cuts and reseals two strands of DNA simultaneously using energy supplied by ATP to create negative supercoiling (Type II topoisomerase). In contrast, Topo I relaxes the negatively supercoiling by cutting and resealing one strand of DNA at a time (Type I topoisomerase).

The gyrase–Topo I pair also acts in concert to relieve the superhelicity generated during replication and transcription, i.e. the local positive supercoiling ahead of the replication forks and transcription bubbles is relaxed by gyrase, and the local negative supercoiling behind the transcription bubbles is compensated by Topo I. Because of these important physiological roles, gyrase and Topo I are basically essential for viability of bacterial cells, although some defects in one of these proteins may be tolerable or suppressed by mutation in the other.

Another topological issue arises at the termination of replication of circular chromosomes and plasmids, i.e. the resolution of the interlocking catenane daughter molecules. Playing this role is another Type II topoisomerase, topoisomerase IV (Topo IV), which is a homolog of gyrase (1,2). Mutations in *parC* or *parE* result in defective decatenation of the circular chromosomes and plasmids and are generally lethal (3–5), although they may be partially suppressed by simultaneous overexpression of both gyrase subunits in *Escherichia coli* (6).

Most bacteria possess both gyrase and Topo IV. A few exceptions are Corynebacteria, *Campylobacter jejuni*, *Deinococcus radiodurans*, *Treponema pallidum and* some Mycobacteria (such as *Mycobacterium leprae*, *Mycobacterium smegmatis* and *Mycobacterium tuberculosis*) (7,8), which lack *parC* and *parE*. Presumably, decatenation of the circular chromosomes in these bacteria is carried out by gyrase. This notion was supported by the demonstration that the gyrase of *M. smegmatis* indeed possesses a strong decatenation activity as well as supercoiling activity *in vitro* (9).

**Figure 1.**
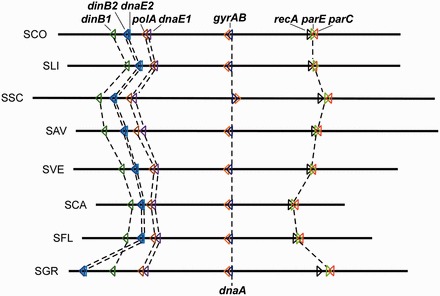
Synteny of *parC* and *parE* genes in *Streptomyces* genomes. Locations and direction of transcription (colored arrowheads) of *parC, parE, gyrAB, dnaA*, *recA*, and the five DNA polymerase genes are indicated on eight sequenced *Streptomyces* chromosomes (oriented according to the *S. coelicolor* chromosome). The chromosomes are centered and aligned at *dnaA*. Chromosome abbreviations: SCO, *S. coelicolor*; SLI, *S. lividans*; SSC, *S. scabiei*; SAV, *S. avermitilis*; SVE, *S. venezuelae*; SCA, *S. cattleya*; SFL, *S. flavogriseus*; SGR, *S. griseus*. The accession numbers and other details of the sequences used are in Supplementary Table S1.

An interesting question arose when linear chromosomes were discovered in some bacteria such as *Borrelia burgdorferi* and *Streptomyces* spp., i.e. do these linear chromosomes require a Type II topoisomerase for decatenation? In theory, replication of linear DNA does not result in catenated molecules, and therefore may not require a decatenase for resolution. However, *parC* and *parE* homologs are present in the chromosomal sequences of these bacteria. In Gram-positive bacteria, *gyrA* and *gyrB* usually form an operon near *oriC*, and *parC* and *parE* lie separately in opposite orientations distally from *oriC*. This is also true for *Streptomyces*. For example, in *S. coelicolor*, the *gyrAB* operon (SCO3873-SCO3874) is near *oriC*, whereas the *parC* and *parE* homologs (SCO5836 and SCO5822) lie in opposite orientations separated by 13 kb on the right arm of the chromosome (Figure 1). Phylogenetic analysis shows that the *parC* and *parE* homologs are grouped in the ParC and ParE branches with those of other bacteria, distinct from the GyrA and GyrB branches, respectively (Supplementary Figure S1). That SCO5822 and SCO5836 of *S. coelicolor* encode the Topo IV subunits was confirmed *in vitro* by Schmutz *et al.* (8) using purified and assembled heterotetrameric topoisomerases. They showed that (SCO5822)_2_(SCO5836)_2_, like other Topo IV (6), possessed both decatenation and relaxation activity, but not supercoiling activity.

Whereas gyrase is essential in *Streptomyces* as well as other bacteria, the role of Topo IV is not clear in *Streptomyces*. It is likely that Topo IV is required for post-replicational decatenation of circular plasmids in *Streptomyces*. In addition, spontaneous circularization of the chromosomes through fusion of the two arms occurs at relatively high frequencies (about 5 × 10^−^^3^ per sporulation cycle) in *Streptomyces* [reviewed in (10)]. One would expect that these circular chromosomes would require a decatenase for post-replicational segregation.

A more interesting question is whether the linear chromosomes and linear plasmids require Topo IV for decatenation in *Streptomyces*. These linear replicons are capped by terminal proteins (TPs) covalently bound at the 5′-ends of the DNA (11). It was shown recently that these TP-capped telomeres interact *in vivo*, resulting in the formation of a circular configuration with negative superhelicity, despite the linearity of these replicons (12). If the telomere–telomere interactions persist throughout and after the completion of replication, the requirement of decatenation would seem imperative.

Moreover, in eukaryotes, despite the linearity of their chromosomes, a type II topoisomerase, Topo II, appears to be required for untangling of the intertwined daughter chromosomes after replication. Mutations that inactivate the decatenation activity of Topo II in yeast result in interlocked chromosomes in S phase (13,14). Topo IV may also perform a similar role for the linear chromosomes in *Streptomyces*.

In this study, we addressed the question whether Topo IV was essential for the linear or circular DNA in *Streptomyces*. We investigated this issue by attempts to delete a Topo IV gene. Our results showed that deletion of *parE* could be achieved on a linear chromosome but not on a circular chromosome, indicating that Topo IV was essential for circular DNA but not for linear DNA in *Streptomyces*. This was confirmed by the ability of the *parE* deletion mutants to support replication of linear plasmids but not circular plasmids. In the *parE* deletion mutants, the linear replicons presumably bypass the requirement of Topo IV through dissociation and reestablishment of the telomere–telomere complex to achieve segregation. Nevertheless, Topo IV was likely important for efficient untangling of the linear daughter chromosomes, because the *parE* deletion mutants exhibited retarded growth and sporulation and temperature sensitivity. Moreover, it was also discovered that Topo IV was involved in resolution of DNA knots formed during site-specific integration of circular plasmids.

## MATERIALS AND METHODS

### Bacterial strains and plasmids

Strains and plasmids used in this study are listed in Table 1. Microbiological and genetic manipulations in *E. coli* and *Streptomyces* were according to Sambrook *et al.* (27) and Kieser *et al.* (26). *Streptomyces* strains were cultured on six solid media, LB (Difco), low-salt LB (LB Lennox, Difco), protoplast regeneration medium R5 (26), SFM (0.2% mannitol, 0.2% soya flour and 0.2% agar) (26), PYM (0.5% peptone, 0.3% yeast extract, 0.3% malt extract, 1% glucose and 2% agar) (28) and DNA (Difco Nutrient Agar), and two liquid media, TSB (0.3% tryptone soya broth powder) and YEME (0.3% yeast extract, 0.5% peptone, 0.3% malt extract, 1% glucose, 34% sucrose and 5 mM MgCl_2_) (26). pLUS355 was constructed from pLUS970 (21) by removing a 3.3-kb *Bcl*I–*Bsm*I fragment containing the *rlr* locus required for replication in linear form and replacing a 0.6-kb *Bcl*I–*Sph*I fragment downstream of *tsr* with multiple cloning site sequence.

**Table 1.** Bacterial strains and plasmids used in this study

Culture/plasmid	Genotype/description	Source/reference
*S. coelicolor*		
M145	Wild type, SCP1^-^ SCP2^-^	(15)
M145/pLUS379	pLUS379 integrated into the chromosome via single crossover	This study
M145ΔparE	M145 containing Δ*parE*::*aac(3)IV* mutation	This study
M145ΔparE/pLUS385	M145ΔparE harboring pLUS385	This study
3456	*Pgl* SCP1^NF^ SCP2^-^	(16)
3456/pLUS379	pLUS379 integrated into the chromosome via single crossover	This study
3456ΔparE	3456 containing Δ*parE*::*aac(3)IV* mutation	Figure 2, this study
3456ΔparE/pLUS385	3456ΔparE harboring pLUS385	This study
*E. coli*		
BW25113/pIJ790	K12 derivative; *araBAD rhaBAD*/λ-RED (*gam bet exo*) *cat araC rep101^ts^*	(17)
ET12567/pUZ8002	*dam*-13::Tn*9 dcm cat tet hsdM hsdR zjj-201*::Tn*10*/*tra neo* RP4	(18)
Plasmids		
pIJ773	*E. coli* plasmid, *aac(3)IV oriT*	(17)
St5B8	*S. coelicolor* cosmid containing spanning *recA* and *parE*	(19)
pLUS379	St5B8 derivative in which *parE* is replaced by the Apr^r^ cassette	This study
pHZ132	*E. coli–Streptomyces* temperature-sensitive shuttle plasmid containing pSG5 ARS, *cos*, *oriT*, *tsr* and *bla*	(20)
pLUS355	Derivative of pLUS970 (21), in which the *rlr* locus is removed and a multiple cloning site is inserted.	This study
pLUS356	Derivative of pLUS970 (21), in which the *Hind*III–*Sfi*I fragment is replaced by a multiple cloning site	Figure 5A, this study
pLUS383	Derivative of pHZ132 (20) containing *tsr* and *bla* and a deletion of the 1.6-kb *HPa*I–*Hin*dIII fragment containing *cos*	This study
pLUS385	pLUS383 derivative containing *parE*	Figure 2A, this study
pIJ702-117	Derivative of pIJ702, *tsr*, *melC* (with a up-regulating promoter)	(22)
pLUS891	Plasmid containing pSLA2 ARS (23) and *tsr* flanked by a pair of 320-bp telomere sequences of SCP1	(24)
pIJ82	pSET152 (25) derivative containing *hyg* replacing *aac(3)IV*	(26)
pIJ82–parE	pIJ82 containing *parE*	Figure 4A, this study

### Construction of *parE* mutants in *Streptomyces*

The gene replacement method based on Gust *et al.* (17) was used to generate the deletion mutants in this study. Basically, the *parE-*disrupting cassette was generated by polymerase chain reaction (PCR) (primers H4-5′ and H4-3′; Supplementary Table S2), which contained a unique priming site annealed to the apramycin-resistant (Apr^r^) cassette from pIJ773 and 36-bp sequences flanking each side of *parE* on the chromosome. This amplified fragment was introduced by transformation into *E. coli* BW25113/pIJ790 harboring a cosmid clone—St5B8 of *S. coelicolor* (19) containing *parE*, and Apr^r^ transformants were selected, which harbored the cosmid with a Δ*parE*::*aac(3)IV* allele (designated pLUS379). pLUS379 was transferred by conjugation from *E. coli* ET12567/pUZ8002 (18) to *S. coelicolor* M145 and 3456 for gene replacement. Apr^r^ exoconjugants were selected. These exoconjugants were kanamycin-resistant (Kam^r^), indicating that they contained integrated pLUS379. Further attempts to isolate kanamycin-sensitive (Kam^s^) Apr^r^ segregants among the exoconjugants to obtain Δ*parE*::*aac(3)IV* mutants failed. For complementation, a 2.6-kb sequence spanning *parE* and 200 bp upstream of it from *S. coelicolor* was inserted into pLUS383, a derivative of the temperature-sensitive plasmid pHZ132 (20), which conferred thiostrepton and viomycin resistance. The resulting plasmid, designated pLUS385, was introduced into *S. coelicolor* by transformation. From thiostrepton-resistant (Thio^r^) exoconjugants, Kam^s^ segregants were obtained that contained the Δ*parE* deletion. Subsequently, loss of pLUS385 was achieved by screening at 40°C.

### Complementation of *parE* mutants

To complement the *parE* mutation in 3456ΔparE and M145ΔparE, the *parE* coding sequence with upstream promoter region (761 bp) was generated by PCR (primers C4-XbaI-5’ and C4-EcoRV-3’; Supplementary Table S2). The amplified fragment was cloned into an integrative plasmid, pIJ82, giving rise to pIJ82–parE. The resulting plasmid was introduced into *E. coli* ET12567/pUZ8002 and further integrated into *S. coelicolor* 3456 and M145 ϕC31 *attB* site via *E. coli–Streptomyces* conjugal transfer. Hygromycin-resistant transconjugants were selected and verified by Southern blotting.

### Microscopy

Aerial mycelium and spore chains were collected on sterile coverslips, inserted in minimal medium containing mannitol for 13 days, according to the methods of Kim *et al.* (8). The coverslips were stained with 5 µg/ml DAPI (4′,6′-diamino-2-phenylindole) in phosphate-buffered saline containing 50% glycerol, and then examined with a fluorescence microscope (Leica DMLB) with 360-nm excitation light and a 425-nm emission filter.

### Phylogenetic analysis

Sixteen bacteria were selected to represent *Streptomyces*, actinobacteria and other bacteria (see Supplementary Table S1 for genomic source information). The orthologs of gyrase and Topo IV from each bacterium were extracted from the Kyoto Encyclopedia of Genes and Genomes database (29), and used for the construction of a phylogenetic tree using the Neighbor-Joining method in Molecular Evolutionary Genetics Analysis (MEGA) software version 5 (30).

## RESULTS

### The Topo IV component, *parE*, was deleted in two steps

We first attempted to delete *parE* (SCO5822) in wild-type *S. coelicolor* M145 using the REDIRECT procedure of Gust *et al.* (17). Cosmid pLUS379 contained a kanamycin resistance gene (*aph*) and a segment of *S. coelicolor* DNA, in which *parE* was replaced by an apramycin-resistance gene [*aac(3)IV*] cassette. Conjugal transfer of the cosmid from *E. coli* to M145 produced Apr^r^ exoconjugants. The insertion of the cosmid in the *parE* region by homologous recombination in these exoconjugants was confirmed by PCR analysis (data not shown). Subsequent attempts to isolate kanamycin-sensitive (Kan^s^) segregants from the M145/pLUS379 exoconjugants, which would have undergone a second crossover and deleted *parE*, failed among more than 600 colonies screened.

We have previously experienced similar difficulties in attempts to delete *polA* (DNA polymerase I) and *recA* from M145, but succeeded with relative ease in 3456 (a strain containing an integrated plasmid SCP1^NF^) (31,32). Thus, we attempted to delete *parE* in 3456 using the same procedure. Apr^r^ 3456/pLUS379 exoconjugants were similarly isolated using pLUS379. However, attempts to isolate Kan^s^ segregants also failed among 450 exoconjugants screened.

**Figure 2.**
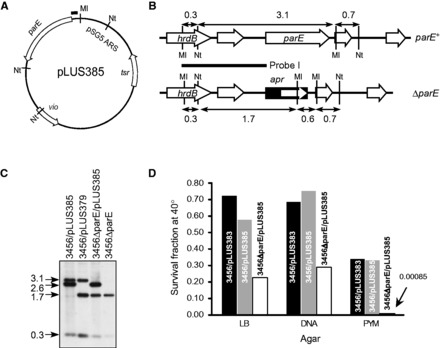
Creation and characterization of temperature-sensitive (*ts*) mutants of *parE* in M145 and 3456. (**A**) Temperature-sensitive plasmid pLUS385 containing *parE* (SCO5822) on the 2.6-kb *Mlu*I (Ml)-*Not*I (Nt) fragment for complementation. *tsr*, thiostrepton resistance gene. The bar on the top indicates the 268-bp sequence that may be hybridized by Probe I (see B). (**B**) Restriction maps of the *hrdB-parE* region on the chromosome of M145 and 3456 (*parE*^+^) and the Δ*parE* mutants. Probe I used in Southern blotting (below) is indicated by the horizontal bar. The *Mlu*I and *Not*I cutting sites are marked, and the sizes of the restriction fragments are indicated in kb. *apr*, apramycin resistance gene. (**C**) Genomic DNA was isolated from the constructed strains, digested with *Mlu*I and *Not*I, and subjected to Southern hybridization using Probe I. The sizes (kb) of the hybridizing fragments are indicated on the left. Representative data of the 3456 series are shown: 3456/pLUS385, 3456/pLUS379, 3456ΔparE/pLUS385, and 3456ΔparE. (**D**) Temperature sensitivity of the mutant strains. The relative plating efficiencies of 3456/pLUS383, 3456/pLUS385, and 3456ΔparE/pLUS385 at 40°C versus 30°C on LB, DNA and PYM agars are represented by the bars. The datum for 3456ΔparE/pLUS385 on PYM is too low to be visible in the chart, and is given as a number.

To check the possibility that *parE* was essential for viability, we introduced a temperature-sensitive plasmid, pLUS385, which contained a viomycin-resistance gene (*vph*), a thiostrepton-resistance gene (*tsr*) and *parE* (Figure 2A), into M145/pLUS379 and 3456/pLUS379. From the Thio^r^ transformants, Kan^s^ segregants were readily isolated at frequencies of approximately 10^−^^1^ in M145/pLUS379 and 10^−^^2^ in 3456/pLUS379. That these Thio^r^ Kan^s^ segregants had suffered deletion of *parE* through double crossovers was confirmed by restriction and hybridization (Figure 2B and 2C). These Δ*parE* mutants still possessed pLUS385 (being Thio^r^), and were designated M145ΔparE/pLUS385 and 3456ΔparE/pLUS385, respectively.

If *parE* was essential for viability of *Streptomyces*, it was expected that M145ΔparE/pLUS385 and 3456ΔparE/pLUS385 would exhibit temperature sensitivity, because replication of the vector (pHZ132) that carried *parE* was defective in replication at elevated temperature (20). Indeed, compared with the control cultures (M145/pLUS385 and 3456/pLUS385), the plating efficiencies of M145ΔparE/pLUS385 and 3456ΔparE/pLUS385 at 40°C were reduced by 60–70% on LB and DNA agar, and by more than two orders of magnitude on PYM agar (Figure 2D).

The colonies that survived the elevated temperature from plating of the M145ΔparE/pLUS385 and 3456ΔparE/pLUS385 spores were analyzed for the presence of pLUS385. If *parE* was essential, it was expected that these surviving cultures would have retained pLUS385. Instead, all these surviving cultures were plasmid-less and Thio^s^ (data not shown). Moreover, these cultures were all Apr^r^, and restriction and Southern hybridization confirmed deletion of the *parE* sequence (data not shown). Therefore, these results indicated that *parE* was not essential for either of these strains, which were designated M145ΔparE and 3456ΔparE, respectively. The initial failure to isolate these deletion mutants directly was most likely due to their retarded growth and sporulation (see following text).

### Δ*parE* exhibited defective growth, and temperature sensitivity

Compared with the wild type, M145ΔparE and 3456ΔparE also grew more slowly at the normal temperature (30°C) on several solid media, particularly R5 and LB agars (Table 2). They grew also very slowly in YEME broth, but normally in TSB broth. We suspected that the poor growth might be correlated to higher osmolality of these media (Table 2 and Supplementary Table S3). This notion was supported by the comparison of LB (containing 10 g/l NaCl) and low-salt (Lennox) LB (containing 5 g/l NaCl), in which the lower salt appeared to benefit the growth of the mutants.

**Table 2.** Growth defect of Δ*parE* mutants

Medium		Solid medium						Liquid medium	
		R5	LB	Low-salt LB	SFM	PYM	DNA	YEME	TSB
Osmolality (mOsm/kg)		815	415	243	147	122	65	1833	312
Growth at 30°C	*parE*^+(^^a^^)^	+++	+++	+++	+++	+++	+++	+++	+++
	Δ*parE*^(^^b^^)^	+	+	++	+++*	++	+++	+	+++
Growth at 42°C	*parE*^+^	+	+++	+++	ND	+++	+++	ND	ND
	Δ*parE*	+	−	+	ND	+	++	ND	ND

^a^M145, 3456, M145ΔparE/pIJ82–parE and 3456ΔparE/pIJ82–parE.

^b^M145ΔparE and 3456ΔparE.

+++, normal growth; ++, slightly reduced growth; +, poor growth; −, no growth; *, retarded sporulation; ND, not determined.

**Figure 3.**
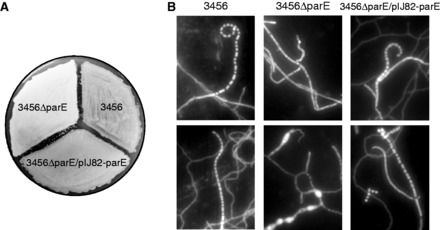
Growth characteristics of the *parE* mutant and the complementation strain. (**A**) 3456, 3456ΔparE, and 3456ΔparE/pIJ82–parE were grown on SFM agar for 4 days at 30°C. 3456ΔparE remained white with aerial hyphae, while the other two strains had produced gray spores. (**B**) 3456, 3456ΔparE, and 3456ΔparE/pIJ82–parE were grown over coverslips on MM containing mannitol for 13 days, and the spores were collected from the coverslips, stained with DAPI and imaged under a fluorescence microscope. Image contrast has been increased for better clarity. More sample photos are in Supplementary Figure S2.

On the medium (SFM) used for conjugation during the construction of the mutants, the vegetative growth of M145ΔparE and 3456ΔparE was comparable with that of the wild-type strains. However, sporulation of the mutants was significantly retarded on this medium. Whereas white aerial hyphae formed in the mutant colonies at about the same time (about 4 days after plating of the spores) as in the wild type, gray spores appeared only sparsely even 10 days after plating (Figure 3A).

The poor sporulation of the mutants was probably due to deficiency in decatenation of the chromosomes during spore formation, where proper partitioning is critical (33). When the 3456ΔparE colonies were examined by DAPI staining under the microscope, the aerial hyphae contained relatively few spores of normal shapes. In the rare spore chains, anucleate spaces (lacking spore-like structure) were often observed (Figure 3B upper panel). The frequency of these anucleate spaces was about 18%. In comparison, the frequencies of anucleate spores in M145 and M145ΔparE/pIJ82–parE were below 1%. In addition, the aerial hyphae of 3456ΔparE often contained very large bulges and extrusions that were packed with excess of DNA (lower panel). This was in contrast to the regularly distributed nucleoids in the aerial mycelial segments and the spore chains in 3456 and 3456ΔparE/pIJ82–parE.

### Δ*parE* mutants were complemented by integrated *parE*

**Figure 4.**
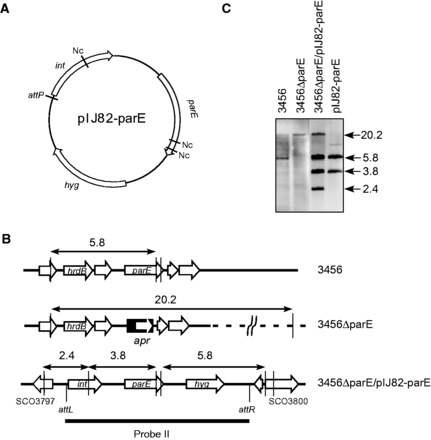
Complementation of the *parE* mutants of 3456 and M145. (**A**) The integrative plasmid pIJ82–parE containing the *parE* coding sequence and 761-bp upstream promoter region. *hyg*, hygromycin resistance gene. *int*, integrase gene of ϕC31 phage. *attP*, ϕC31 attachment site. Nc, *Nco*I site. (**B**) The restriction maps of the *hrdB-parE* region on the chromosome of 3456, 3456ΔparE, and 3456ΔparE/pIJ82–parE (harboring the integrated pIJ82–parE). Probe II used in Southern blotting (below) is indicated by the horizontal bar. The *Nco*I cutting sites are indicated by the vertical lines, and the sizes of the restriction fragments are indicated in kb. (**C**) Confirmation of integration of *parE* complementation. Genomic DNA was digested with *Nco*I, and subjected to Southern hybridization using the Probe II. The sizes (kb) of the hybridizing fragments are indicated on the right.

To complement the Δ*parE* mutation, the *parE* coding sequence with its upstream promoter region (761 bp; from the termination codon of the upstream gene to the initiation codon of *parE*) was inserted into an integrative plasmid, pIJ82 (26), and the resulting plasmid, pIJ82–parE (Figure 4A), was introduced into M145ΔparE and 3456ΔparE. Hygromycin-resistant transformants were isolated, and integration of the plasmid into the ϕC31 *attB* site was confirmed by restriction and hybridization (Figure 4B). These complementation strains, designated M145ΔparE/pIJ82–parE and 3456ΔparE/pIJ82–parE, grew as well as wild type on solid media and in liquid media tested (Table 2). The retarded sporulation on SFM and abnormal nucleoid distribution displayed by M145ΔparE and 3456ΔparE were also eliminated (Figure 3B). These results confirmed that the observed growth defects of M145ΔparE and 3456ΔparE were due to deletion of *parE*.

### Δ*parE* mutants cannot maintain circular plasmids

The ability to tolerate the Δ*parE* mutation in M145 and 3456 indicated that these linear chromosomes in *Streptomyces* do not require Topo IV for resolution. This is intriguing in view of the fact that the linear chromosomes and plasmids form a circular configuration (herein termed ‘pseudo-circle’) *in vivo* through telomere–telomere association in *Streptomyces* (12). There are two possible bypass mechanisms for the post-replicational untangling of the daughter replicons of these pseudo-circles: (*i*) gyrase may substitute for Topo IV for the untangling function, and (*ii*) the interacting telomeres of these linear replicons may transiently dissociate from each other for the untangling. These hypotheses may be tested with a circular plasmid. The first bypass mechanism would allow a circular plasmid to propagate in the deletion mutants, whereas the second mechanism would not.

**Figure 5.**
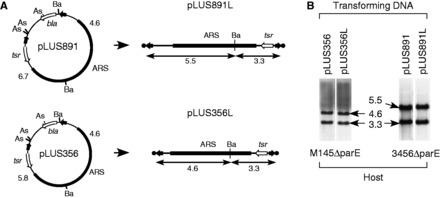
Inability of circular plasmids to replicate in the Δ*parE* mutants, M145ΔparE and 3456ΔparE. (**A**) Circular plasmids pLUS356 and pLUS891 (left) used for transformation and the linear versions, pLUS356L and pLUS891L, generated in the transformants (right). *bla*, beta-lactamase gene; *tsr*, thiostrepton resistance gene; ARS, autonomously replicating sequence of pSLA2; filled arrows, telomeres of the *S. lividans* chromosome (on pLUS356) or SCP1 plasmid (on pLUS891); filled circles, TPs. As, *Ase*I site; Ba, *Bam*HI site. The sizes (kb) of the *Bam*HI fragments of the linear DNA are indicated. (**B**) M145ΔparE and 3456ΔparE were transformed by pLUS356 or pLUS891. Genomic DNA was isolated from the Thio^r^ transformants, digested with *Bam*HI, and subjected to Southern blotting using the transforming DNA as the respective probes. Representative transformants are shown. The sizes (kb) of the hybridizing fragments are indicated.

First, we tested pIJ702-117, which contained *tsr* and the *melC* operon (22). This plasmid transformed M145 and 3456 at ‘normal’ frequencies, but failed to produce any transformants in M145ΔparE and 3456ΔparE. Next, we tested two circular plasmids, pLUS356 and pLUS891, both of which included a linear plasmid sequence (Figure 5A) (24). These plasmids may appear and replicate as free linear molecules (with TP-capped telomeres) in the transformants under certain conditions. M145 and 3456 were transformed by these two plasmids at ‘normal’ frequencies, but M145ΔparE and 3456ΔparE were transformed at frequencies of about two orders of magnitude lower. The transformants were examined for the presence of plasmids. Interestingly, all of the 21 pLUS356 transformants (9 in 3456ΔparE and 12 in M145ΔparE) and 26 pLUS891 transformants of 3456ΔparE examined contained only the linear versions of these plasmids (i.e. ‘pLUS356L’ and ‘pLUS891L’; Figure 5B), and no circular plasmids. In contrast, the M145 and 3456 transformants contained only circular but no linear plasmids. For comparison, we tested pLUS355, a variant of pLUS970 that lacked the *rlr* locus required for replication in linear form and thus can replicate only in circular form in *Streptomyces*. No Thio^r^ transformants could be produced in M145ΔparE or 3456ΔparE.

Finally, another transformation was performed using a mixture of 3456 and 3456ΔparE protoplasts at comparable concentrations of colony forming units (Table 3). *Ase*I-linearized pLUS356 DNA produced 221 transformants of 3456 and 120 transformants of 3456ΔparE, representing transformation frequencies of 9.6 × 10^−^^5^ and 2.3 × 10^−^^5^, respectively. The plasmids present in these transformants were all linear (pLUS356L; not shown). In contrast, pLUS355 DNA produced 594 Thio^r^ transformants of 3456, but none of 3456ΔparE.

**Table 3.** The *parE* knockout mutant can maintain the linear plasmid (pLUS356L) but not circular plasmid (pLUS355)

Treatment	3456^a^	3456ΔparE^a^	Total^a^
No. cfu regenerated	2.3 × 10^6^	5.2 × 10^6^	7.5 × 10^6^
No. pLUS355 transformants	594	0	594
No. pLUS356/*Ase*I transformants	221	120^b^	341

^a^Total transformants were scored by thiostrepton resistance; 3456ΔparE was scored by the number of Apr^r^ transformants; 3456 was scored by subtracting the total number of transformants by that of Apr^r^ transformants.

^b^Twenty-two transformants were checked by Southern hybridization, and all showed the existence of the linear plasmids.

These results showed that Topo IV was required for the maintenance of circular plasmids, but not linear plasmids, in *Streptomyces*, and that gyrase could not functionally substitute for Topo IV in the decatenation of the circular plasmids.

### Δ*parE* mutant cannot maintain a circular chromosome

Based on these findings, it was likely that Topo IV was also required for maintenance of circular chromosomes in *Streptomyces*. This could be tested by investigating spontaneous chromosome circularization in the Δ*parE* mutants. The linear chromosomes of *Streptomyces* are highly unstable, undergoing spontaneous deletions and fusions of terminal sequences at relatively high frequencies (11). These terminal deletions are often very long, up to 1 Mb (34), and are readily detected by the loss of chloramphenicol resistance genes located in the terminal region—e.g. *cmlR1* (SCO7526) and *cmlR2* (SCO7662) located 322 and 179 kb, respectively, from the right end of the *S. coelicolor* chromosome. Such chloramphenicol-sensitive (Cml^s^) mutants arise spontaneously at frequencies in the range of 10^−^^3^ to 10^−^^2^ after a sporulation cycle.

We reasoned that the lack of Topo IV in the Δ*parE* mutants should eliminate the appearance of circularized chromosomes in them, and thus reduce the appearance of Cml^s^ mutants. This was confirmed by comparing the spontaneous Cml^s^ mutation rates of 3456 and 3456ΔparE. In one sporulation cycle, 3456 produced Cml^s^ mutants at a frequency of 0.28% (9/3200), and 3456ΔparE produced no Cml^s^ mutants among 3200 colonies examined (<0.03%). Complementation of the *parE* gene in 3456ΔparE restored the spontaneous Cml^s^ mutation rate to 0.36% (8/2100). The results supported the notion that Topo IV was essential for the viability of circular chromosomes (Fisher’s exact test, *P*-value = 0.00388).

### Topo IV is involved in plasmid integration

**Figure 6.**
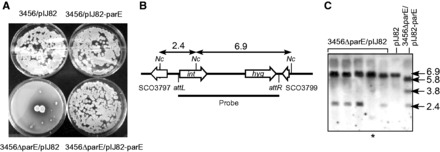
Faulty site-specific plasmid integration in the Δ*parE* mutants. (**A**) Transformation of 3456ΔparE with pIJ82 is defective. 3456 and 3456ΔparE were transformed with pIJ82 and pIJ82–parE, and scored for Hyg^r^ transformants after 10 days of incubation. (**B**) Restriction maps of the integration site. The phage attachment sites (*attL*, *attR*), the *Nco*I restriction sites, and the sizes (kb) of the expected restriction fragments are indicated. (**C**) Integration of pIJ82 DNA in 3456ΔparE. Genomic DNA was isolated from several Hyg^r^ transformants of 3456ΔparE, digested with *Nco*I, and subjected to electrophoresis and Southern hybridization using pIJ82 DNA as the probe. Representative samples are shown. The sizes (kb) of the hybridizing fragments are indicated. In one transformant (marked with the asterisk), the pattern of the hybridizing fragments was atypical, suggesting that the plasmid might have integrated into a secondary site.

During the introduction of pIJ82–parE into M145ΔparE and 3456ΔparE for complementation, parallel experiments were performed using pIJ82 as controls. Whereas pIJ82–parE transformed 3456ΔparE mutants at about the same efficiency as it did 3456, unexpectedly, pIJ82 transformed 3456ΔparE at frequencies about two orders of magnitude lower than it did 3456. The pIJ82 transformants of 3456ΔparE appeared very late. Whereas the pIJ82 transformants of 3456 (or the pIJ82–parE transformants of either strains) appeared within 4 days, the appearance of the pIJ82 transformants of 3456ΔparE was delayed by several days, and the sizes of the colonies varied widely (Figure 6A). Nevertheless, these transformants contained the integrated pIJ82, as shown by restriction and Southern hybridization (Figure 6B and C), and, on subsequent subculturing, they grew at about the same rate as 3456ΔparE, and did not exhibit heterogeneous colony size. Thus, it appeared that the obstacle lies within the initial transformed cells, which, once resolved, did not pose any hindrance in the progeny cells. We interpreted the obstacle to be DNA knots produced during site-specific recombination, which could not be resolved by Topo IV in 3456ΔparE, and caused lethality.

## DISCUSSION

Topo IV is essential for segregation of circular daughter chromosomes and plasmids in bacteria. Mutations in the coding genes have been found to cause either lethality or thermosensitivity in bacteria with circular chromosomes (4,35). Initially, we also failed to delete *parE* directly in M145 and 3456, and the preliminary results were reported previously (36). However, in this study, we showed that in *Streptomyces* cultures with a linear chromosome, the chromosomal *parE* could be deleted in the presence of a complementing copy on a plasmid, and the removal of the plasmid produced Δ*parE* mutants that exhibited growth and sporulation deficiencies. The deficiencies explain the earlier difficulties in isolating the mutants directly by screening for segregants through double crossovers.

The successful isolation of the Δ*parE* mutants demonstrated that Topo IV is not essential for wild-type *Streptomyces* with a linear chromosome. This is interesting because the linear chromosomes and linear plasmids have been demonstrated to exit in circular configuration through telomere–telomere interactions (12). If the telomere–telomere interactions persist through replication, ‘pseudo-catenanes’ would be produced, which would have to be resolved for proper segregation. Thus, in the Δ*parE* mutants, it is likely that dissociation of the telomere–telomere complex occurs during or after replication to allow the decatenation of the pseudo-catenane.

Such dissociation of the telomere–telomere complex has been proposed as a possible mechanism for solving another post-replicational segregation problem for linear replicons posed by intramolecular telomere–telomere associations. It was pointed out that if the ‘old’ TPs on the parental strains of the linear plasmid or chromosome remain associated with each other through replication, and the new TPs capping the daughter DNA become associated with each other, a pseudo-dimeric (Möbius strip-like) structure would result (12,36). These pseudo-dimers may be resolved by exchanging the interacting TPs, i.e. the ‘old’ TPs switching to associate with the ‘new’ ones at the opposing telomere. It is likely that this partner changing mechanism is also used to resolve the pseudo-catenanes at least in the Δ*parE* mutants.

In some actinobacteria (such as Corynebacteria and *M. smegmatis*), which lack *parC* and *parE* in their genomes, the role of decatenation of their circular replicons must fall on gyrase. However, in *Streptomyces*, gyrase is unlikely to perform decatenation, because circular plasmids and circular chromosomes cannot be maintained in the Δ*parE* mutants.

Why did the Δ*parE* mutants exhibit poor growth and sporulation? There are two plausible non-mutually exclusive possible reasons. First, it may be due to low efficiency of decatenation of the pseudo-catenanes by dissociation of the telomere–telomere complexes. The dissociated telomeres must reconnect in correct topology to achieve separation. Second, it could be that Topo IV is also important in untangling daughter linear chromosomes, as shown in yeasts (37–40). The *Streptomyces* chromosomes are typically 6–10 Mb, significantly larger than the chromosomes of *Saccharomyces cerevisiae* (2.2–0.2 Mb) and *Schizosaccharomyces pombe* (5.7–3.5 Mb). The anomaly of nucleoid size and distribution in the hyphae of the *parE* mutants (Figure 3B) supports these notions.

The poor growth of the Δ*parE* mutants was more severe on rich media and at high temperature. Presumably, the faster chromosomal replication in the cultures under these conditions demand timely post-replicational resolution and partitioning of the chromosomes before the next round of replication is completed. Alternatively, the higher osmotic pressure in the richer media (Supplementary Data and Supplementary Table S3) may be an important factor. Such elevated sensitivities to rich media, osmotic pressure and/or thermosensitivity have been often observed in topoisomerase mutants of bacteria (41–43).

It is intriguing that some actinobacteria with circular chromosomes lack Topo IV, whereas *Streptomyces* spp. with linear chromosomes possess Topo IV, which is not absolutely required. The linear chromosomes of *B. burgdorferi* also possess Topo IV. In this case, the telomeres are hairpinned instead, and there is no evidence for telomere–telomere interactions in this bacterium.

Why do *Streptomyces* spp. possess a decatenase that is not absolutely required? There are at least three possible advantages: (i) it decatenates better than the proposed transient dissociation of the telomere–telomere complex, (ii) it allows harboring of circular plasmids and (iii) it allows the survival of mutants in which the chromosomes have circularized. The last point is intimately related to the extremely high occurrence of chromosome circularization in *Streptomyces*, a puzzle that is poorly understood in terms of molecular mechanism and evolutional significances. As we have shown here, without Topo IV, no circular chromosomes can survive.

The Δ*parE* mutants also exhibited low transformation efficiencies of pIJ82, which integrates into the *Streptomyces* chromosomes at a specific *attB* site. Site-specific recombination involving a circular DNA molecule may produce knots, which are harmful for cells if not efficiently removed. Zechiedrich *et al.* (44) have shown that the products of site-specific recombination by λ Int or Tn*3* resolvase were decatenated by Topo IV, not gyrase. López *et al.* (45) also showed Topo IV to be the topoisomerase that unknots DNA during replication. Presumably, Topo IV is also important for unknotting in *Streptomyces*.

The pIJ82 transformants of 3456ΔparE appeared in very few numbers very slowly and sporadically. In these transformants, the knots had presumably been removed by fortuitous events at various times before cell death, which allowed the hyphae to resume growth. One possibility was the occurrence of a double-strand break in the knot region followed by repair, which removed the knots by chance. In any case, on replating, these transformants grew like 3456ΔparE without wide variations in colony size. This supports the notion that the Topo IV was important only during the site-specific recombination stage when knots were formed.

## SUPPLEMENTARY DATA

Supplementary Data are available at NAR Online.

## FUNDING

National Science Council [101-2311-B-010-009, 101-2321-B-010-019]; and a National Professorship from the Ministry of Education, R. O. C., (to C. W. C.). Funding for open access charge: National Science Council, R. O. C. [101-2311-B-010-009, 101-2321-B-010-019].

*Conflict of interest statement*. None declared.

## Supplementary Material

Supplementary Data
