# Supplementary material for: Topoisomerase IV is required for partitioning of circular chromosomes but not linear chromosomes in Streptomyces
Source: Nucleic Acids Res. 2013 Aug 31;41(22):10403–13. doi: 10.1093/nar/gkt757 (PMC3905888; doi:10.1093/nar/gkt757)
Supplement: Supplementary Data [file supp_41_22_10403__index.html]

Topoisomerase IV is required for partitioning of circular chromosomes but not linear chromosomes in Streptomyces — Topoisomerase IV is required for partitioning of circular chromosomes but not linear chromosomes in Streptomyces — Supplementary Data 

# Topoisomerase IV is required for partitioning of circular chromosomes but not linear chromosomes in *Streptomyces*

## Supplementary Data

files

**Files in this Data Supplement:**

- Supplementary Data - pdf file
