# Supplementary material for: Topoisomerase IV is required for partitioning of circular chromosomes but not linear chromosomes in Streptomyces
Source: Nucleic Acids Res. 2013 Aug 31;41(22):10403–13. doi: 10.1093/nar/gkt757 (PMC3905888; doi:10.1093/nar/gkt757)
Supplement: Supplementary Data [file supp_gkt757_nar-01062-m-2013-File008.pdf]

Supplementary Figure S1

GyrA-ParC

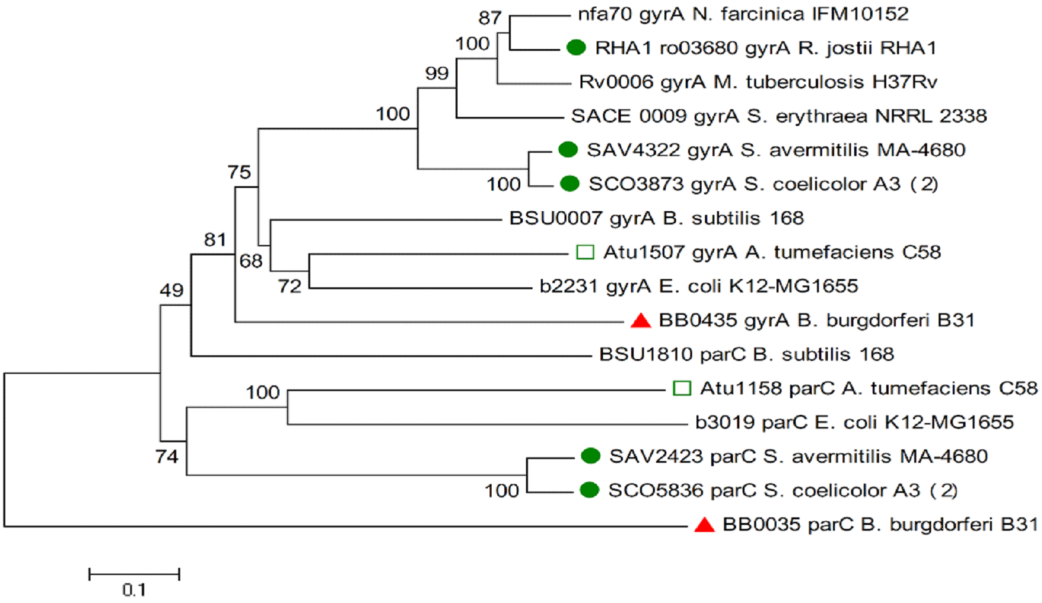

GyrB-ParE

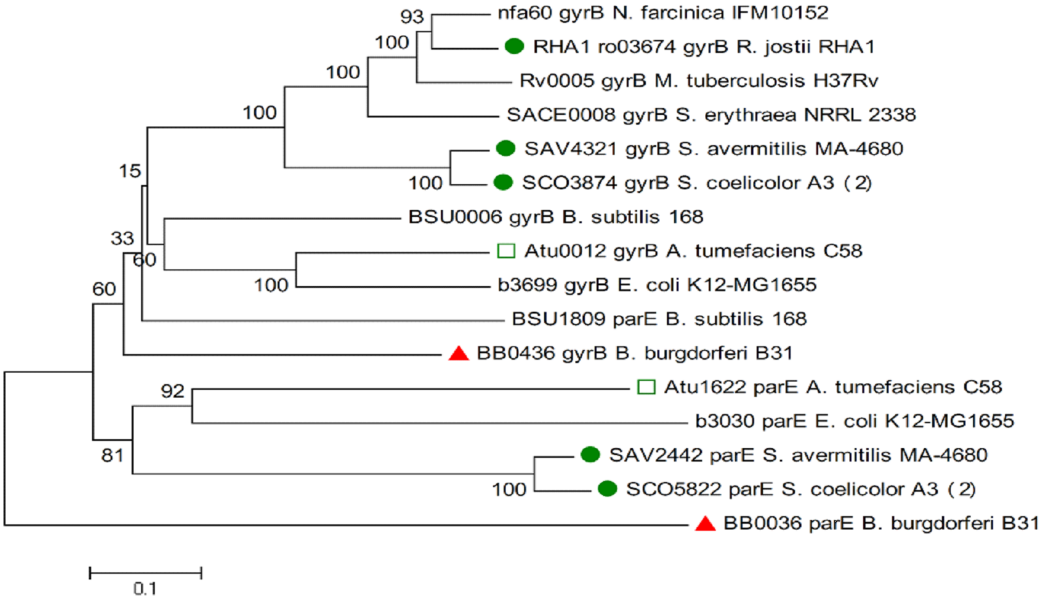

Figure S1. The phylogenetic trees of DNA gyrase (GyrA and GyrB) and Topo IV (ParC and ParE). The orthologs of DNA gyrase and Topo IV subunits in five Streptomyces (filled circles), five other actinobacteria (open circles), five other bacteria (open squares), and Methanosarcina acetivorans (as the out group) were used to constructed the two phylogenetic trees using neighbor-joining method in MEGA5 software. Bootstrap values (with 1,000 replicates) are shown at the branching points. The scale bar represents a 5% sequence divergence. The sources of the sequences are listed in the Table S1 of Supplementary Materials. (A) The GyrA/ParC tree. (B) The GyrB/ParE tree.

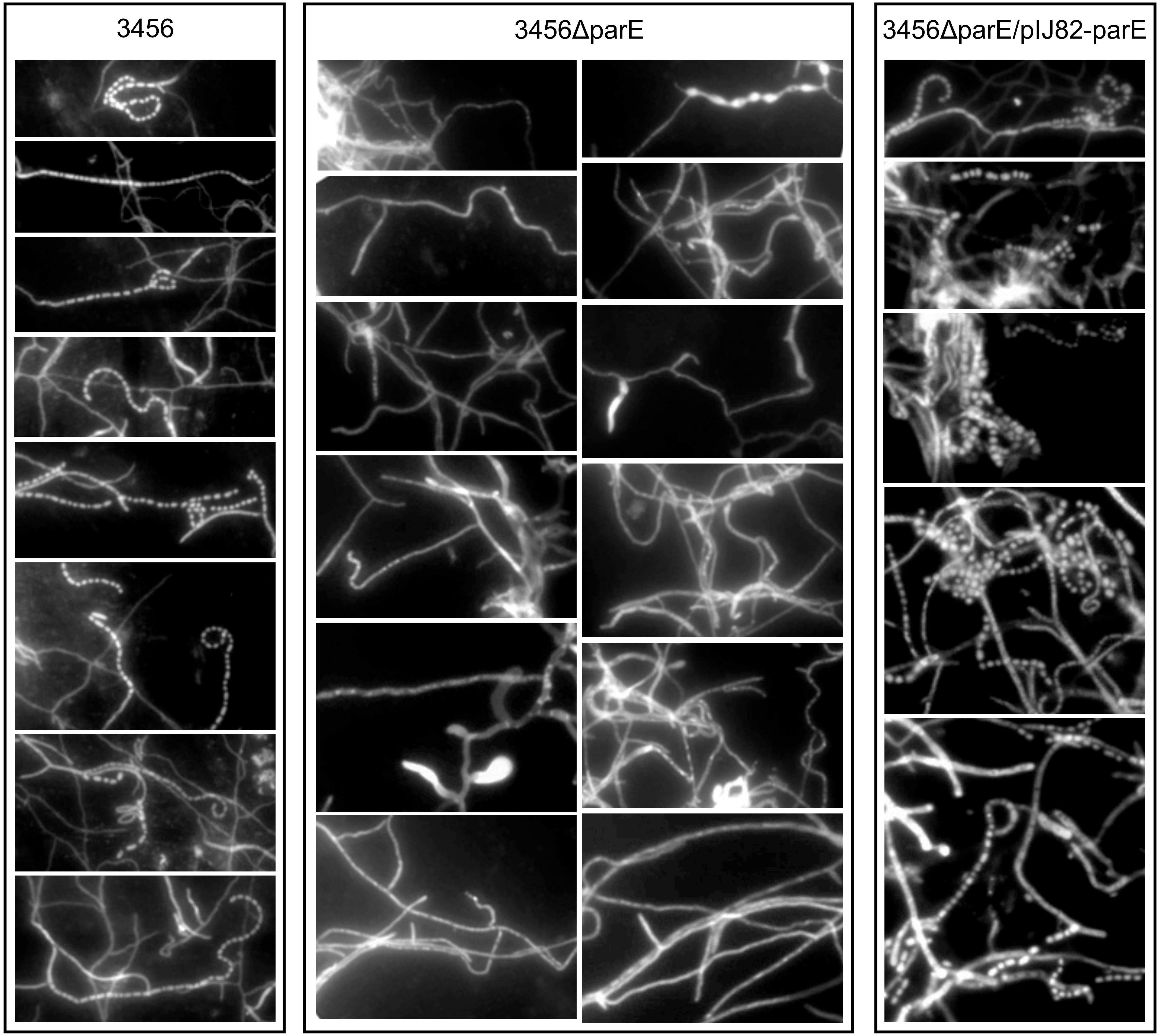

Figure S2. Spore chain morphology of the *parE* mutant.

3456, 3456ΔparE, and 3456ΔparE/pIJ82-parE were grown over coverslips on MM containing mannitol for 13 days, and the spores were collected from the coverslips, stained with DAPI and imaged under a fluorescence microscope. Image contrast has been increased for better clarity.

Table S1. Selected bacterial chromosomes and their orthologs of DNA gyrase or TOPO IV used in the phylogenetic analyses in Figure S1.

Species	Taxonomy	Topology[1]	Size (Mb)	Accession number	GyrA	ParC	GyrB	ParE
Streptomyces avermitilis MA-4680	Actinobacteria	L	9.03	NC_003155	SAV_4322	SAV_2423	SAV_4321	SAV_2442
Streptomyces coelicolor A3(2)	Actinobacteria	L	8.67	NC_003888	SCO3873	SCO5836	SCO3874	SCO5822
Rhodococcus jostii RHA1	Actinobacteria	L	7.80	NC_008268	RHA1_ro03680	ND[2]	RHA1_ro03674	ND
Mycobacterium tuberculosis H37Rv	Actinobacteria	C	4.41	NC_000962	Rv0006	ND	Rv0005	ND
Saccharopolyspora erythraea NRRL 2338	Actinobacteria	C	8.21	NC_009142	SACE_0009	ND	SACE_0008	ND
Nocardia farcinica IFM 10152	Actinobacteria	C	6.02	NC_006361	nfa70	ND	nfa60	ND
Agrobacterium tumefacien C58	γ-proteobacteria	C<br>L	2.84<br>2.07	NC_003062<br>NC_003063	Atu1507	Atu1158	Atu0012	Atu1622
Bacillus subtilis 168	Firmicutes	C	4.21	NZ_ABQK00000000	BSU00070	BSU18100	BSU00060	BSU18090
Borrelia burgdorferi B31	Spirochaetes	L	0.91	NC_000950	BB0435	BB0035	BB0436	BB0036
Escherichia coli K12-MG1655	γ–proteobacteria	C	4.64	NC_000913	b2231	b3019	b3699	b3030

[1] L, linear; C, circular. [2] 'ND', not detected.

Table S2

Designation	Sequence
H4-5’	GGGGCCCATCTGAAATTCAGCGAGGAGCGAACCGCGTGattccggggatccgtcgacc
H4-3’	TCACGCTCCCGGGCCCGGTCCGACGGCTGAGCTGCCTCAtgtaggctggagctgcttc
T4-MluI-5’	ACGCGTCGGGTTCGCGCAGTCGGTC
T4-3’	CCTCCGTCAGCGGAAGAAGG
5822 up-5’	CCACTACCTCCCGGCCACC
5822 up-3’	CGCTGAATTTCAGATGGGGCC

The lower case letters indicate the sequences complementary to pIJ773 template.

Table S3. Osmotic pressures of different media

Medium	Osmolality (mOsm/kg)*
YMD	82.7 ± 0.2
STE buffer	806.3 ± 1.2
LB	415.3 ± 1.5
YEME	1832.7 ± 2.5
R2YE	814.7 ± 1.5
0.3P buffer	620.3 ± 2.5
TSB	312 ± 2.0
10.3% sucrose	489.7 ± 2.5
NMMP	329.3 ± 0.7
DNB (DNA-agar)	65.0 ± 0.0
SFM (MS)	147.3 ± 0.6
PYM	122.3 ± 0.6

* Mean of three independent measurements followed by the standard deviations.
